# The meta-analysis of beef cattle body weight prediction using body measurement approach with breed, sex, and age categories

**DOI:** 10.5455/javar.2023.j718

**Published:** 2023-12-31

**Authors:** Frediansyah Firdaus, Bayu Andri Atmoko, Endang Baliarti, Tri Satya Mastuti Widi, Dyah Maharani, Panjono Panjono

**Affiliations:** 1 Research Center for Animal Husbandry, National Research and Innovation Agency, Cibinong Science Center, Bogor, Indonesia; 2 Department of Animal Production, Faculty of Animal Science, Universitas Gadjah Mada, Yogyakarta, Indonesia; 3 Department of Animal Genetic, Faculty of Animal Science, Universitas Gadjah Mada, Yogyakarta, Indonesia

**Keywords:** Body weight, correlation, meta-analysis, beef cattle, body measurement

## Abstract

**Objective:**

The aim of the study was to use a meta-analysis to identify the correlation between linear body measurements, including body length (BL), wither height (WH), heart girth (HG), and body volume (BV), and body weight in beef cattle by breed, sex, and age as categories.

**Materials and methods:**

These results can be used as a method for predicting beef cattle body weight. This study used systematic review and meta-analysis guidelines to create a checklist. The first stage was searching for papers relevant to the study objectives. The second stage was searching using the keywords beef cattle, body weight, body measurement, and correlation. The third stage was reviewing the title and abstract. The fourth stage was abstracting information from selected papers, and the last stage was tabulating data.

**Results:**

The results from this study were obtained, and 32 papers were eligible for the meta-analysis stage. The correlation between linear body measurement and body weight of beef cattle showed that HG (*r* = 0.88) and BV (*r* = 0.97) were significantly (*p* < 0.05) different compared to BL (*r* = 0.74) and WH (*r* = 0.72). The correlation between HG and body weight, and the categorization of cattle breeds showed significantly (*p* < 0.05) different results. The correlation between BV and body weight of cattle according to breed categories showed results that were not significantly (*p* > 0.05) different, while age was significantly (*p* < 0.05).

**Conclusion:**

In conclusion, to predict beef cattle body weight, it is necessary to use HG or BV, with breed, sex, and age of cattle as categories.

## Introduction

Monitoring beef cattle productivity, which includes body weight, plays an important role [1]. Furthermore, body weight can be used as a reference for measuring the amount of feeding [2], buying and selling [3], and supporting fattening programs [4]. Subsequently, body weight is often measured using manual digital scales, which require the cattle to move across the scale. This can be stressful for the livestock and requires extra labor and time. Finally, farmers often rely on visual assessment to determine livestock body weight, which is subjective and whose accuracy depends on experience [5,6]. Firdaus et al. [7] reported that farmers were able to predict the body weight of cattle with accuracy ranging from 77.71% to 91.57%, varying based on age, education, and farming experience.

As an alternative, linear body measurements, including body length (BL), wither height (WH), heart girth (HG), and body volume (BV) regression modeling, can be used to predict the body weight of cattle. However, the existing study is generally limited to certain breeds, sexes, and ages, and the number of samples used during modeling varies [8,9]. Therefore, the prediction accuracy of cattle body weight still varies. Tyasi et al. [10] reported that the correlation between body weight and linear body measurements was HG (*r* = 0.76), while Chico-Alcudia [11] reported a correlation between HG and body weight (*r* = 0.98). Moreover, the correlation analysis between body measurement and the body weight of cattle in predicting the body weight of cattle to provide an overview has never been performed globally. This significance lies in its ability to determine the global applicability of the formula for predicting cattle body weight. Modeling should be performed for each specific breed, sex, and age. The meta-analysis method can provide a more in-depth description of determining body measurement variables and the categorization recommendations used. Meta-analysis is a method for inferring the results of several studies using certain statistical methods. Meta-analysis aims to integrate the quantitative results of selected studies into a numerical estimate, which summarizes all study results [12].

Based on the description above, the aim of the study was to use a meta-analysis to analyze the correlation between BL, WH, HG, and BV with body weight in beef cattle by breed category, sex, and age of cattle.

## Materials and Methods

### Ethical approval

This research is an article review in the form of a meta-analysis, so it does not use ethical approval.

### Meta-analysis

We conducted this study following the systematic review and meta-analysis guidelines [13]. We conducted the systematic review method in several stages. The first stage was searching for published papers relevant to the study objectives using the Google Scholar, CABI, and Science Direct databases from 2002 to 2022. The second stage was searching using the keywords beef cattle, body weight, body measurement, and correlation. The keywords used, defined based on the PICO concept, are population (P): beef cattle; intervention (I): correlation; comparator (C): body measurement; and outcome (O): body weight. The third stage was reviewing the title and abstract. The fourth stage was abstracting information from selected papers, and finally, the last stage was tabulating data using study category, year of publication, country, breed, sex, age, number of samples, and correlation coefficient. We obtained the correlation coefficient value by correlating HG, BL, WH, and BV with beef cattle body weight. Subsequently, BV was a calculation with a tube volume approach. BV = BL × {*π* × (LD/2*π*)²}. BV uses cm^3^ units; when converted to liters, then BV(dm^3^) = BL × {*π* × (LD/2*π*)²}/1,000 = (BL × LD²)/(4,000π). Specific tabulation of breed, sex (male and female), and age of cattle (A1 (1–12 months), A2 (>12–24 months), and A3 (>24 months).

**Figure 1.**
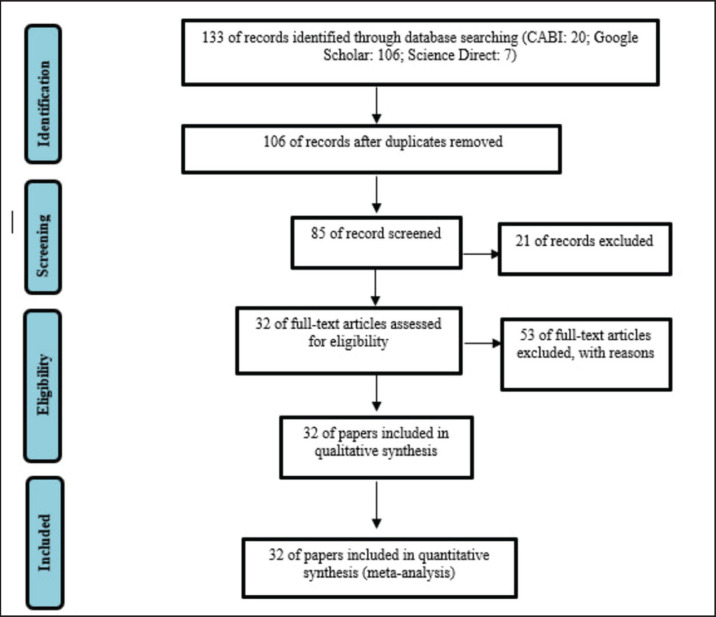
The systematic review and meta-analysis flow chart using CABI, Google Scholar, and Science Direct database.

The papers selected for this study are required to meet the following criteria: The papers selected for this study must meet the following criteria: (1) they must be published in Indonesia or English; (2) they must use research material in the form of beef cattle; (3) they must report the breed, sex, age, number of samples, correlation coefficient, year of publication, and country for each experiment; and (4) they must use simple linear regression. The analysis with systematic review guidelines identified 133 papers (Fig. 1). We obtained 106 papers after eliminating the duplicates. After checking relevant titles and abstracts, we obtained 85 papers. After examining the availability of data on the number of samples and the level of the correlation coefficient, as well as the availability of categorization by breed, sex, and age, we obtained 32 published papers for the meta-analysis stage.

**Table 1.** Database of studies of predicting body weight of beef cattle used in meta-analysis.

No	Authors	Year	Country	Breed	Sex	Age	*N* (head)
1	Putra et al. [16]	2014	Indonesia	Aceh	M, F	A2	79
2	Gunawan and Jakaria [17]	2010	Indonesia	Bali	M	A1	278
3	Niam et al. [18]	2012	Indonesia	Bali	F	A2, A3	80
4	Hikmawaty et al. [19]	2018	Indonesia	Bali	F	A2	44
5	Jakaria et al. [20]	2019	Indonesia	Bali	M, F	A2	68
6	Zurahmah and The [21]	2011	Indonesia	Bali	M	A2	31
7	Paputungan et al. [22]	2018	Indonesia	Bali	F	A1, A2, A3	394
8	Tisman and Putra [23]	2015	Indonesia	Bali, Bali Cross	M	A3	116
9	Yanto et al. [24]	2021	Indonesia	Brahman Cross	F	A3	32
10	Hafiz et al. [25]	2014	Malaysia	Brakmas	F	A1	363
11	Ahmed et al. [26]	2019	Sudan	Butane	F	A2	34
12	Bahashwan [27]	2014	Oman	Dhofari	M, F	A1	72
13	Yakubu [28]	2010	Nigeria	Fulani	F	A2, A3	83
14	Ige et al. [29]	2015	Nigeria	Fulani	M, F	A3	45
15	Haq et al. [30]	2020	Indonesia	Jabres	M, F	A1	123
16	Sawanon et al. [31]	2011	Thailand	Kamphaeng Saen	M	A3	504
17	Przysucha et al. [32]	2012	Poland	Limousine	F	A3	419
18	Prihandini et al. [33]	2020	Indonesia	Madura	M	A1, A3	198
19	Abud et al. [34]	2018	Brazil	Nellore	F	A2	56
20	Laya et al. [35]	2020	Indonesia	Ongole Crossbreed	F	A3	340
21	Paputungan [36]	2015	Indonesia	Ongole Crossbreed	F	A3	363
22	Ersi et al. [37]	2018	Indonesia	Ongole Crossbreed	M	A1	30
23	Sarwono et al. [38]	2019	Indonesia	Ongole Crossbreed	F	A1	97
24	Paputungan et al. [39]	2013	Indonesia	Ongole Crossbreed	F	A3	363
25	Sahu et al. [40]	2016	India	Sahiwal	F	A3	193
26	Siddiqui et al. [41]	2015	Pakistani	Sahiwal	M	A3	350
27	Bene et al. [42]	2007	Hungary	Simmental	F	A3	40
28	Suliani et al. [43]	2017	Indonesia	Simpo	M	A2, A3	90
29	Musa et al. [44]	2012	Sudan	Sudanese Kenana	M	A3	75
30	Putra [45]	2020	Indonesia	Sumba Ongole	M, F	A2	58
31	Abdelhadi & Babiker [46]	2012	Sudan	Western Baggara	M	A3	274
32	Sakar et al. [47]	2020	Turkey	Yerli Kara	M, F	A1	407

M = male, F = female, *N* = total head; A1 = 1–12 months, A2 = >12–24 months, A3 = >24 months.

### Statistical analysis

According to the meta-analysis, this study performed this part. To measure the effect size, this study used the coefficient correlation. To assess the value of the heterogeneity of the estimated effect size, this study used Cochran’s *Q* test. The *I*^2^ was between 0% and 100%. The substantial heterogeneity between studies is indicated by an *I*^2^ value greater than 50% [14]. A random effects model was applied to award relative weights to each study in the meta-analysis. Finally, the cumulative effect size was transformed back to the correlation coefficient. After that, the results of the meta-analysis were interpreted and reported. Estimates of average true correlations were declared significant at *p* < 0.05. Meta-analysis was performed using OpenMEE software [15].

## Results

The study obtained data from 12 countries, including Sudan, Indonesia, Poland, Oman, Thailand, Hungary, Brazil, India, Pakistan, Nigeria, Malaysia, and Turkiye. The majority of the research data on predicting cattle body weight in this study came from Indonesia (56.25%) and Sudan (9.4%), with the categories of cattle being Bali cattle (21.9%) and Ongole Crossbreed (15.6%) (Table 1). All selected papers include complete data on the number of samples and the correlation coefficient value, and they utilize a simple linear regression approach where the independent variable influences the dependent variable. Selected papers have specific breed, sex, and age data. According to the meta-analysis on the relationship between body measurement and body weight in beef cattle, HG and BV differed significantly (*p* < 0.05) from BL and WH (Table 2).

HG and prediction of body weight in cattle using HG with categorization based on cattle breed showed significantly different results based on subgroup meta-analysis. Cattle are divided into three categories based on the cumulative effect size value (Table 3). The strength of the correlation for a variable body measurement of HG only reaches 0.86–0.91 if there is no classification based on breeds. Once categorized, the chance of strengthening the correlation can reach above 0.95. Table 3 displays the correlation strengthening above 0.95, including Jabres, Dhofari, Sahiwal, Fulani, and Sumba Ongole cattle.

**Figure 2.**
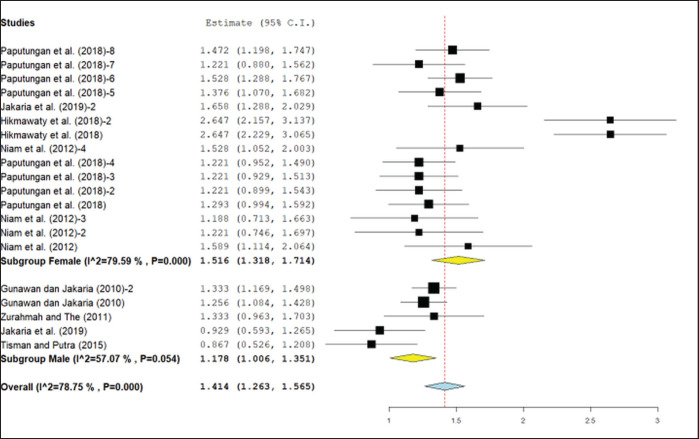
Meta-analysis of the correlation between HG and body weight of Bali cattle with sex categorization.

**Figure 3.**
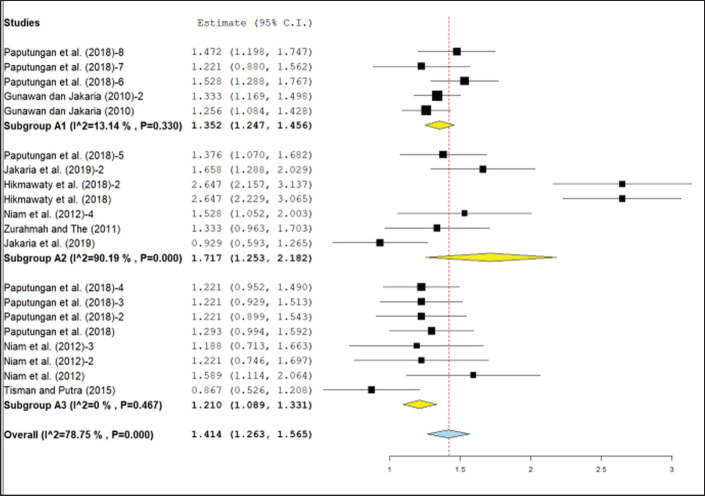
Meta-analysis of the correlation between HG and body weight of Bali cattle with age categorization. A1 = 1–12 months; A2 = >12–24 months; A3 = >24 months.

The results of the meta-analysis showed that sex and age were not significantly different (Table 3). However, due to differences between breeds, there was a need to investigate sex differences in certain categories of cattle breeds. In Bali cattle, the correlation between HG and body weight, by sex (Fig. 2) and age (Fig. 3), showed significantly different results, based on a subgroup meta-analysis. The correlation coefficient value for male cattle was 0.83 (0.76–0.81), while for female cattle, it was 0.91 (0.87–0.94).

The results of the meta-analysis of Bali cattle based on age (Fig. 3) resulted in significantly different cumulative effect size values, where A1 and A2 category cattle were significantly different from A3 category cattle. The correlation coefficient value for cattle A1 is 0.87 (0.85–0.90), A2 is 0.94 (0.85–0.97), and A3 is 0.84 (0.80–0.87). It is believed that the A1 and A2 categories of cattle experienced growth following a straight line, while the A3 cattle had a more sloping linear line.

## BV

The correlation between BV and body weight of cattle according to breed categories showed results that were not significantly (*p* > 0.05) different, while at age they were significantly (*p* < 0.05) different (Table 4), with a strong correlation value. This suggests that BV has the potential to produce an accurate body weight prediction compared to using single variables such as BL and WH.

**Table 2.** Meta-analysis of the correlation of various body measurements with the body weight of beef cattle.

Variable	Linear body measurement	Body weight (kg)	Coefficient correlation			Heterogenity	*N* (head)
			Estimate	Lower	Upper		
BL	145.1 cm	258.8	0.74^a^	0.67	0.79	94.51%	4,401
WH	115.8 cm	263.7	0.72^a^	0.63	0.79	95.32%	3,344
HG	112.5 cm	245.5	0.88^b^	0.86	0.91	93.34%	5,162
BV	254.5 dm^3^	288.4	0.97^b^	0.97	0.98	74.02%	757

^a,b^Different letters in the diagram indicate significant differences based on the meta-analysis subgroups. *N* = total sample; BL = body length; WH = wither height; HG = heart girth; BV = body volume.

**Table 3.** Meta-analysis of the correlation between HG and body weight of beef cattle in the category of breed, sex, and age.

Variable	HG (cm)	Body weight (kg)	Coefficient correlation			Heterogenity	*N* (head)
			Estimate	Lower	Upper		
Breed							
Aceh	118.1	129.4	0.89^b^	0.77	0.95	71.33	79
Ongole crossbreed	170.3	436.4	0.89^b^	0.81	0.93	95.58	1,193
Bali	139.5	190.8	0.89^b^	0.85	0.92	78.75	931
Dhofari	119.6	135.9	0.96^c^	0.92	0.98	51.7	72
Kamphaeng saen	154.1	314.7	0.89^b^	0.84	0.92	78.64	504
Fulani	92.3	116.0	0.94^c^	0.92	0.96	0	128
Jabres	116.1	103.7	0.96^c^	0.95	0.97	0	123
Sudanese kenana	149.6	243.8	0.52^a^	0.32	0.68	0	75
Sumba ongole	154.5	262.9	0.95^b^	0.89	0.98	48.08	58
Sex							
Male	137.1	199.2	0.86	0.80	0.91	95.39	2,902
Female	149.6	292.6	0.89	0.87	0.92	90.94	2,305
Age							
A3 (>24 months)	167.1	367.2	0.86	0.81	0.90	95.26	3,384
A2 (>12–24 months)	135.4	193.1	0.92	0.86	0.95	87.09	491
A1 (1–12 months)	111.7	110.4	0.89	0.85	0.93	90.13	1,287

^a,b,c^Different letters in the diagram indicate significant differences based on the meta-analysis subgroups.* N* = total sample.

**Table 4.** Meta-analysis of correlation between BV and body weight of beef cattle by breed and age with female sex.

Variable	BV (dm^3^)	Body weight (kg)	Coefficient correlation			Heterogenity	*N* (head)
			Estimate	Lower	Upper		
Breed							
Ongole crossbred	348.5	435.5	0.97	0.96	0.98	82.21	363
Bali	195.8	196.4	0.97	0.96	0.98	69.94	394
Age							
A3	331.6	379.8	0.97^a^	0.96	0.98	55.97	553
A2	132.9	135.9	0.97^a^	0.95	0.98	-	44
A1	63.5	82.7	0.98^b^	0.98	0.99	69.24	160

^a,b^Different letters in the diagram indicate significant differences based on the meta-analysis subgroups. *N* = total samples; A1 = 1–12 months; A2 = >12–24 months; A3 = >24 months.

## Discussion

The prediction of the body weight of cattle with linear body measurements can be applied according to the expected goals and efficiency. Subsequently, fewer independent variables are needed, but with a large increase in accuracy, it becomes easier to apply body weight prediction formulas in the field. HG and BV, with strong correlation results according to the outcome of the meta-analysis, are recommended in predicting the body weight of cattle. There is a strong positive correlation between chest circumference and body weight for predicting body weight, which was observed in previous studies for various breeds, ages, and sexes of cattle. This is in accordance with the reports from Washaya et al. [48] and Gudeto et al. [49]. HG is the best variable for predicting the body weight of cattle compared to other body sizes in various breeds, ages, and sexes of cattle. Similar studies were reported by Ashwini et al. [50]. HG can be used to predict the body weight of crossbred cattle age group-wise. HG and BV have the potential to be used as independent variables for predicting body weight in cattle. The higher the dimension used, the better the correlation coefficients. BL and WH use only one dimension, namely length or height. While the heart girth uses a two-dimensional approach, the BV uses a three-dimensional approach, combining three elements: length, symbolized by BL, and area of the base, symbolized by the HG approach.

The prediction model for cattle body weight is influenced by the breed and sex of the cattle. Between cattle, there is more genetic diversity compared to cattle in other regions. Various environmental conditions can influence these differences, such as pasture conditions, water availability, temperature and humidity, and disease resistance [51]. The categorization of sex and age in Bali cattle, which is the breed of cattle with the largest percentage in this meta-analysis study, showed significantly different results. Sex affects the growth of body tissues and, therefore, the composition of body tissues. Subsequently, sex differences in muscle weight distribution develop with the growth of livestock. Bulls have a higher muscle-to-bone ratio than male and female calves. This is because bulls produce heavier carcasses at certain fat levels, and therefore, they appear to have a greater drive for muscle growth. Cano et al. [52] reported that the growth of cattle after 2 years of age did not follow a linear curve. It shows that categorization based on nationality, gender, and age is needed to obtain a higher value of the correlation coefficient, determination coefficient, and accuracy.

## Conclusion

The variables of HG and BV resulted in a higher correlation coefficient than BL and WH. Furthermore, categorization by breed, sex, and age should be carried out to produce a higher correlation coefficient value. It could be concluded that for better prediction of beef cattle body. It is necessary to use HG or BV, with categories of breed, sex, and age of cattle.

## List of abbreviations

A, age; BL, body length; WH, wither height; HG, heart girth; BV, body volume.
